# 25-Year Old Male with Pleural Thickening

**DOI:** 10.4103/0970-2113.44126

**Published:** 2008

**Authors:** Abhilasha Ahuja, D Gothi, G Amonkar, J M Joshi

**Affiliations:** Department of Respiratory Medicine, T. N. Medical College & B Y L Nair Hospital, Mumbai 400 008; Department of Pathology, T. N. Medical College & B Y L Nair Hospital, Mumbai 400 008

## Abstract

The growth of some of the adenocarcinomas is virtually identical to that of malignant mesothelioma, also known as pseudomesotheliomatous adenocarcinoma of lung. Their differentiation on the basis of histopathology can pose diagnostic difficulties; hence immunohistochemistry and electron microscopy may be required for further differentiation.

## INTRODUCTION

Pseudomesotheliomatous adenocarcinoma is an uncommon variant of peripheral adenocarcinoma first described by Harwood et al in 1976. It usually presents with signs of pleural thickening. Hence it may be a diagnostic challenge in patients in whom malignancy is least suspected. Further, even if a diagnosis of malignancy is made on histopathology, it may be confused with epithelial variety of mesothelioma. This case is being reported to emphasize the presence of this entity and modalities which can be used to make the exact diagnosis.

## CASE HISTORY

**Fig. 1:**
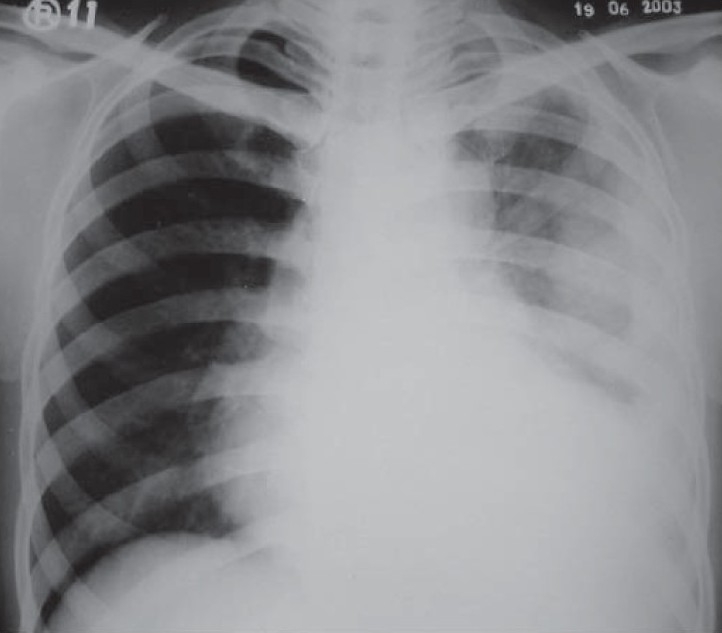
Chest radiograph shows inhomogeneous opacification of left hemithorax suggestive of left sided pleural thickening.

**Fig. 2:**
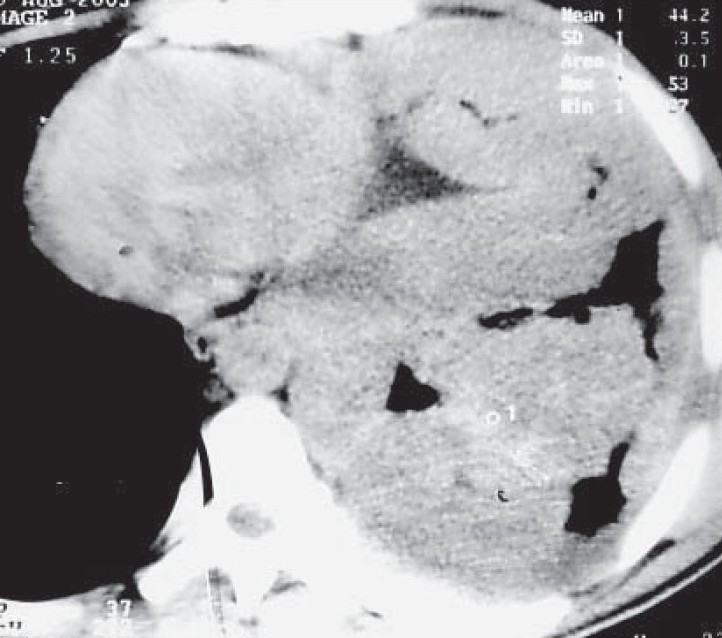
Computerized tomography of the thorax reveals a left sided diffuse pleural thickening and large soft tissue mass arising from thoracic and mediastinal pleura.

**Fig. 3:**
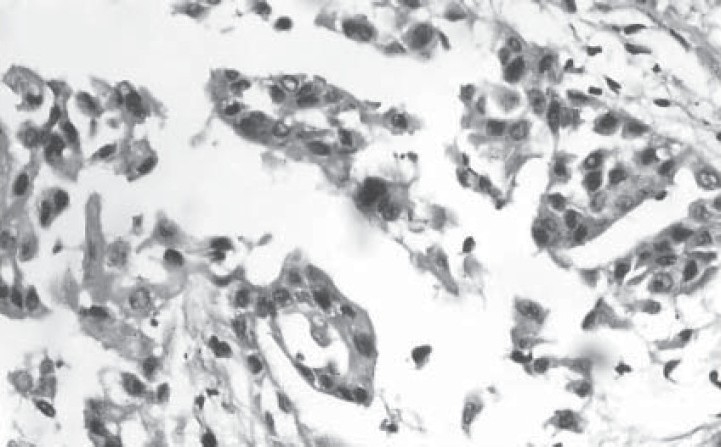
Histopathological examination of the biopsy specimen showing tumor cells in sheets and glandular arrangement with hyperchromatic nuclei (Haematoxylin and eosin, X 40).

A 25-year-old, non-smoker, male presented with dull aching left sided chest pain, dry cough and exertional dyspnoea for past two months. He was being treated with antituberculosis drugs over past two months for left sided pleural effusion. He denied history of exposure to organic or inorganic dust. There was no evidence of digital clubbing or lymphadenopathy. Examination was unremarkable except loss of volume, dull note and decreased auscultatory sounds over entire left hemithorax. Chest radiograph (fig. 1) shows inhomogeneous opacification of left hemithorax suggestive of left sided pleural thickening. Computerized tomography (fig. 2) of the thorax reveals a left sided diffuse pleural thickening and large soft tissue mass arising from thoracic and mediastinal pleura. No hilar or mediastinal lymphadenopathy was seen. Pleural fluid analysis showed an exudate, negative for malignant cells, bacteria, fungi and mycobacteria. Ultrasonography of abdomen and bone scan was normal. CT guided biopsy of the pleural mass under high power field is shown in fig. 3.

Histopathological examination of the biopsy specimen shows (fig 3) tumor cells arranged in papillary fronds, sheets and glandular structures with hyperchromatic nuclei suggestive of either adenocarcinoma or epitheloid variety of mesothelioma. Further, immunohistochemical markers were done to establish the diagnosis. Our biopsy specimen showed diffuse positivity for cytokeratin (CK) and epithelial membrane antigen (EMA), fluffy positivity for Carcinoembryonic antigen (CEA) and thin membrane positivity for mesothelial antigen-HBME-1, thus favoring the diagnosis of adenocarcinoma.

### Diagnosis

Peripheral adenocarcinoma of the lung with extensive pleural involvement also called as pseudomesotheliomatous adenocarcinoma of the lung (stage −3b).

## DISCUSSION

Peripheral adenocarcinoma typically manifests as a nodule with retraction of overlying pleura and an expansile growth pattern that destroys and displaces adjacent lung parenchyma. The borders of tumor may be rounded, lobulated, or poorly defined. The lobulation reflects the histological heterogeneity of lung cancer whereas ill-defined borders may be related to invasion of adjacent lung, fibrosis or interstitial oedema. The growth of some these adenocarcinomas are virtually identical to that of malignant mesothelioma, also known as pseudomesotheliomatous adenocarcinoma of lung.^1^ It is an uncommon variant of peripheral adenocarcinoma first described by Harwood et al in 1976. The few reported cases in literature indicate that the lesion occurs predominantly in men in fifth and sixth decades of life and in second or third decade of life in HIV positive individuals. 2 But its occurrence in third decade of life in HIV negative patient as seen in our case is rare. Other differential diagnoses for pleural based mass with diffuse pleural thickening include solitary fibrous tumor of pleura, malignant mesothelioma, soft tissue sarcomas, malignant fibrous histiocytoma, fibromyxoma, metastatic spindle cell carcinoma of lung and kidney, pseudomesotheliomatous angiosarcoma.

Distinction between pseudomesotheliomatous adenocarcinoma and epithelial variety of mesothelioma may be difficult. Histochemical stains, immunohistochemical markers and electron microscopy can facilitate the distinction between the two. Histochemical stains include Periodic acid Schiff stain after diastase digestion (PAS-D) and Alcian blue stains. PAS-D stains neutral mucin, which is produced by adenocarcinomas. Alcian blue stains acid mucin that is produced by epithelial mesotheliomas. Further, immunohistochemical staining can be done to differentiate between the two. Adenocarcinomas immunoreact with glycoproteins (epithelial markers) like CEA, Ber EP4, B72.3, Cluster of differentiation (CD) 15, and BG-8 whereas mesotheliomas do not react with glycoproteins. Mesotheliomas show a thick, brush border like staining pattern with HBME-1 while adenocarcinoma do not stain with HBME-1 or show a cytoplasmic or thin membrane pattern of staining^3^. Currently there is no immunohistochemical stain, which is consistently positive in mesotheliomas and negative in adenocarcinomas, for example- CK and EMA are expressed in both the tumors. Thus, while adenocarcinoma can be diagnosed by positive staining for of the CEA, CD-15, Ber EP4 or B72.3, the immunohistochemical diagnosis of mesothelioma is one of exclusion^4^. Recently, Ordonez et al have recommend a panel of four markers for mesothelioma (two positive and two negative) from calretinin, cytokeratin5/6 or WT1 for the positive markers and CEA, MOC-31, B72.3, Ber-EP4 or BG-8 for negative markers.^5^ Electron microscopy can also help in distinction; adenocarcinomas have short and thick microvilli as compared to mesotheliomas, which have very long, thin microvilli.^6^

Our patient was referred to oncology for further management. Chemotherapy has an established role in the therapy of Stage-3b non-small cell lung carcinoma; randomized clinical trial data demonstrate improved median and long term survival when antineoplastic agents are used as part of multimodality approach. However, the optimal sequence for various modalities and best chemotherapy regimen remains to be defined.^7^

## BOOK REVIEW

Oxygen Therapy (2nd ED), Edited by S.K. Jindal and R Agarwal (p. 377) Publishers: Jaypee Brothers Medical Publishers (P) Ltd., New Delhi 2008 Price Rs. 295/-

The book on Oxygen Therapy is a comprehensive monograph which includes chapters starting with the history of oxygen to oxygen storage and supply. Most of the chapters are contributed by the Faculty and the postdoctoral fellows of the department of Pulmonary and Critical Care Medicine of the Postgraduate Institute of Medical Education and Research, Chandigarh.

The greatest strength of this monograph lies in its inclusiveness of all aspects of medical oxygen. The chapters on applied physics of gases and respiratory physiology lay the foundations to understand the basic principles which govern the gas exchange. The following chapter on blood gases, acid base balance, blood gas monitoring and hypoxemia are in a logical sequence to the basic physiology. Interpretation of blood gases, often considered as a tricky issue by the postgraduate students as well as the practicing physicians, has been carefully handled. Several of the difficult terms and conditions have been defined in a lucid manner.

The most useful chapters which one finds useful in routine clinical practice are related to the therapeutic use of oxygen for both pulmonary and non-pulmonary disorders. It also shows that some of the traditional indications for the use of oxygen may not be scientifically solid. The chapter on use of oxygen in specific situations such as in the intensitve care units, during air travel, on high altitude and in diving medicine, are brief but very informative. Similarly, the discussion on long term oxygen therapy, hyperbaric oxygen and alternate oxygen carriers provide an useful introduction to the issues of great importantce.

It is almost a must-read book for all the physicians, intensivists, pulmonologists and postgraduate students of medical specialities. It is like a reference document on medical oxygen. The one deficiency which one sometimes feels is the relative paucity of bibliography and the absence of referenced citations. But that does not in any way affect the quality and readability of the book.
